# Genetic manipulation of reptilian embryos: toward an understanding of cortical development and evolution

**DOI:** 10.3389/fnins.2015.00045

**Published:** 2015-02-24

**Authors:** Tadashi Nomura, Wataru Yamashita, Hitoshi Gotoh, Katsuhiko Ono

**Affiliations:** ^1^Developmental Neurobiology, Kyoto Prefectural University of MedicineKyoto, Japan; ^2^Japan Science and Technology Agency, PRESTOKawaguchi, Japan; ^3^Department of Biophysics, Graduate School of Science, Kyoto UniversityKyoto, Japan

**Keywords:** amniotes, reptiles, cortex, *in ovo* electroporation, *ex vivo* culture, evolution

## Abstract

The mammalian neocortex is a remarkable structure that is characterized by tangential surface expansion and six-layered lamination. However, how the mammalian neocortex emerged during evolution remains elusive. Because all modern reptiles have a homolog of the neocortex at the dorsal pallium, developmental analyses of the reptilian cortex are valuable to explore the origin of the neocortex. However, reptilian cortical development and the underlying molecular mechanisms remain unclear, mainly due to technical difficulties with sample collection and embryonic manipulation. Here, we introduce a method of embryonic manipulations for the Madagascar ground gecko and Chinese softshell turtle. We established *in ovo* electroporation and an *ex ovo* culture system to address neural stem cell dynamics, neuronal differentiation and migration. Applications of these techniques illuminate the developmental mechanisms underlying reptilian corticogenesis, which provides significant insight into the evolutionary steps of different types of cortex and the origin of the mammalian neocortex.

## Introduction

The mammalian cerebral cortex is a remarkable brain structure that is responsible for intricate social behaviors and intelligence. The cerebral cortex is characterized by tangential expansion of its surface area, which is particularly enhanced in the primate and human neocortex, and a six-layered laminar structure composed of multiple types of excitatory and inhibitory neurons (Nieuwenhuys, 1994; Kriegstein et al., 2006; Defelipe, 2011; Lui et al., 2011). The basic frameworks of these unique characteristics are accomplished by the dramatic increase in the number of neural stem/progenitor cells and massive irruption of distinct types of neurons, followed by the coordinated migration of differentiated neurons during embryogenesis. Recent advances of developmental neurobiology have illuminated the molecular mechanisms that govern these complicated cellular events during corticogenesis (Campbell, 2005; Flames and Marin, 2005; Dehay and Kennedy, 2007; Molyneaux et al., 2007; Kumamoto and Hanashima, 2014).

On the contrary, the origin and evolutionary process of the mammalian cortex remain elusive. Phylogenic and paleontological evidence indicated that the forerunners of the mammalian lineage diverged from the common ancestors of amniotes at approximately 300 million years ago (Carroll, 1988; Ruta et al., 2003, 2013). Other lineages of amniotes have also diverged into several unique animal groups that include the descent of extant reptiles (Ruta et al., 2003). In recent years, numerous fossil records have been identified from Paleozoic and Mesozoic sediments, which provided significant information on the process of amniote diversification. Three-dimensional tomographic analyses of fossil endocasts suggested that the size of the mammalian cerebral cortex has increased rapidly in accordance with the dependence of olfactory and somatosensory information (Quiroga, 1979; Rowe et al., 2011); however, histological architectures of the ancestral cerebral cortex remains unknown, preventing us from tracing how the cerebral cortex has specifically evolved in the mammalian lineage.

Ontologically, the cerebral cortex is derived from the dorsal pallium (DP), which develops in the dorsal part of the telencephalon in all vertebrate species (Northcutt, 1981; Puelles et al., 2000; Cheung et al., 2007; Aboitiz, 2011). Despite of developmental homology to the cerebral cortex, the DP in non-mammalian amniotes forms in distinct manners: a three-layer lamination is constructed in the reptilian DP, whereas nuclear slabs are formed in the avian DP (Medina and Reiner, 2000; Heyers et al., 2003; Jarvis et al., 2005; Striedter, 2005). Phylogenetically, aves are included in reptiles (Nomura et al., 2013b; Xu et al., 2014), but here we will use the term reptiles to specifically mean “non-avian reptiles” that include lizards, geckoes, turtles and crocodiles. Because reptiles occupy a unique evolutionary position within amniotes, developmental analyses of the reptilian cortex illuminate commonalities and divergence of developmental programs, thus providing significant insights into the origin of the mammalian cerebral cortex. Previous studies identified unique features of reptilian corticogenesis, such as an outside-in pattern of neuronal migration (Goffinet et al., 1986, 1999; Tissir et al., 2003; Aboitiz and Zamorano, 2013), a difference of layer-specific cell types produced in the reptilian dorsal pallium (Reiner, 1991, 1993), a difference regarding the existence of intermediate progenitors (Charvet et al., 2009; Medina and Abellan, 2009), and lower rates of neurogenesis compared to mouse and other mammalian species (Nomura et al., 2013a). However, modern experimental techniques have not been applied to the analyses of reptilian corticogenesis, largely because of several technical difficulties in collection and manipulation of embryos. First, most reptilian species exhibit seasonal reproduction; thus, a large number of embryos at the desired stages are not constantly available. For example, common lizards/geckoes such as *Lacerta trilineata, Anolis carolinensis*, or *Eublepharis macularius* are frequently used as a model animal in comparative developmental biology (Goffinet et al., 1986; McLean and Vickaryous, 2011; Eckalbar et al., 2012; Sanger et al., 2012). The females of these species produce a limited number of eggs after bleeding. Second, unlike chicken, most reptilian species lay soft-shell eggs, which hampers *in ovo* manipulation of embryos. Although a few pioneering works have reported *in ovo* gene delivery or *ex ovo* culture with snake, lizard and turtle embryos (Nagashima et al., 2007; Matsubara et al., 2014; Tschopp et al., 2014), detailed protocols on embryonic manipulation for reptiles have not been provided.

Here, we describe a method of embryonic manipulation techniques for two reptilian species: the Madagascar ground gecko (*Paroedura pictus*) and the Chinese softshell turtle (*Pelodiscus sinensis*). Surgical techniques on developing reptilian embryos enable us to utilize various experimental approaches. We established the introduction of exogenous genes into the reptilian cortex by *in ovo* electroporation. Furthermore, we developed an *ex ovo* culture system for gecko and turtle embryos, which remarkably increased accessibility to the embryos and improved the efficiency of gene introduction. Successful manipulation techniques of non-mammalian embryos are valuable for studies of the evolutionary developmental biology of the cerebral cortex.

## Animals and detailed protocols of new technique

### Madagascar ground gecko

**Figure 1:**
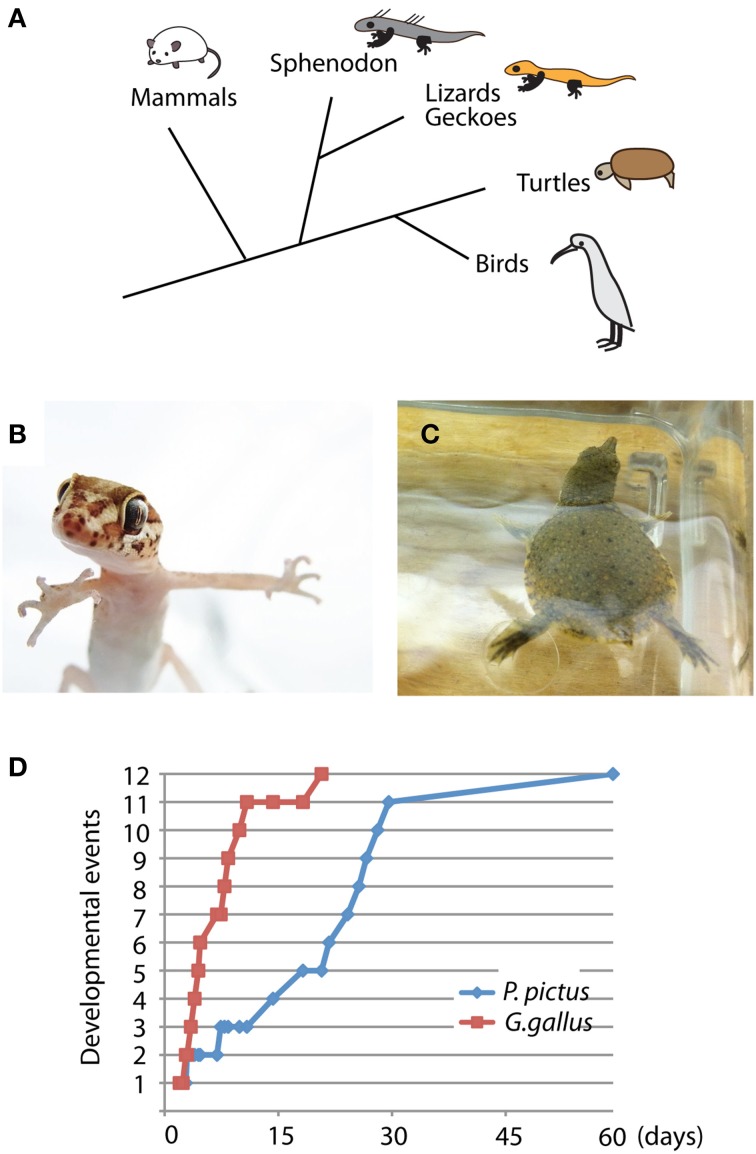
**Unique characteristics of Madagascar ground gecko and Chinese softshell turtle**. **(A)** Phylogenic position of the gecko and turtle among amniotes. Lepidosaurs include sphenodon, snake, lizard and gecko, whereas archosaurs include turtle, crocodile and bird. **(B,C)** Young individuals of Madagascar ground gecko (*Paroedura pictus*) **(B)** and Chinese softshell turtle (*Pelodiscus sinensis*) **(C)**. **(D)** Developmental rates of *Paroedura pictus* and *Gallus gallus* (chick). Equivalent developmental stages are based on limb bud and cranial morphology (Wise et al., 2009). Representative developmental events include 1: hindlimb bud develops, 2: hindlimb bud becomes larger than forelimb bud, 4: autopodium develops discrete paddle shape, 5: zeugopodium and stylopodium become distinct, 6: digits develop, 9: phalanges develop, 10: claws develop, 11: scale formation and pigmentation, and 12: hatching. Detailed staging criteria are described in Wise et al. (2009).

The Madagascar ground gecko (*P. pictus*) is a ground crawling gecko that commonly lives on Madagascar Island. Gecko, lizard, snakes and Tuatara (Sphenodon) are included in the group of lepidosaurs (Figure 1A). The adult size of *P. pictus* is approximately 15–20 cm, and the gecko is easy to handle and breed in captivity (Figure 1B). After mating of a pair of male and female geckoes, a female produces 1 or 2 clutches every 10–20 days and continues to lay eggs for several months (Nomura et al., 2013a,b). Embryonic staging of the gecko has been established by Noro et al., who determined that the gecko embryogenesis proceeds much slower than chicken embryogenesis (Figure 1D) (Noro et al., 2009; Wise et al., 2009). The gecko does not exhibit temperature-dependent sex determination. The embryo hatches approximately 60 days after oviposition and begins to catch small insects within a few days after the first molting. To feed the geckoes, various sizes of crickets were purchased from a local breeder (Tsukiyono farm, Gunma, Japan) and dusted with mineral supplements (calcium and vitamin D) to prevent rickets. To collect embryos, 4 pairs of wild-type geckoes (total 8 animals) were first obtained from a local store (Kansai Reptile Pro, Osaka, Japan) and maintained in our laboratory (28°C, 12 h of light and dark cycles, 50–60% humidity). More than 100 eggs were obtained from 4 females bred for 6 months.

### Chinese softshell turtle

Turtle embryos have been used for anatomical and developmental studies since the nineteenth century (Tokita and Kuratani, 2001). The Chinese softshell turtle (*P. sinensis*) is a freshwater-living turtle that is widely distributed in eastern and southeastern Asia (Figure 1C). The adult size of the turtle reaches over 30 cm in carapace length, and sexual maturity takes approximately 5–6 years. Because the turtle exhibits seasonal reproduction, we could obtained fertilized eggs from a local breeder (Daiwa-Yoshoku, Saga, Japan) in the summer from the beginning of June to the end of August. Sex determination is not dependent on temperature. The developmental stages of the turtle have been established by a previous report (Tokita and Kuratani, 2001). Embryogenesis takes approximately 60 days, and newborn turtles begin foraging after consuming the remaining abdominal yolk. All experimental procedures for reptilian captivity and embryonic manipulation were approved by the experimental animal committee of Kyoto Prefectural University of Medicine (M23-272), and were performed in accordance with the relevant guidelines of the committee.

### Manipulation and electroporation of reptilian embryos

**Figure 2:**
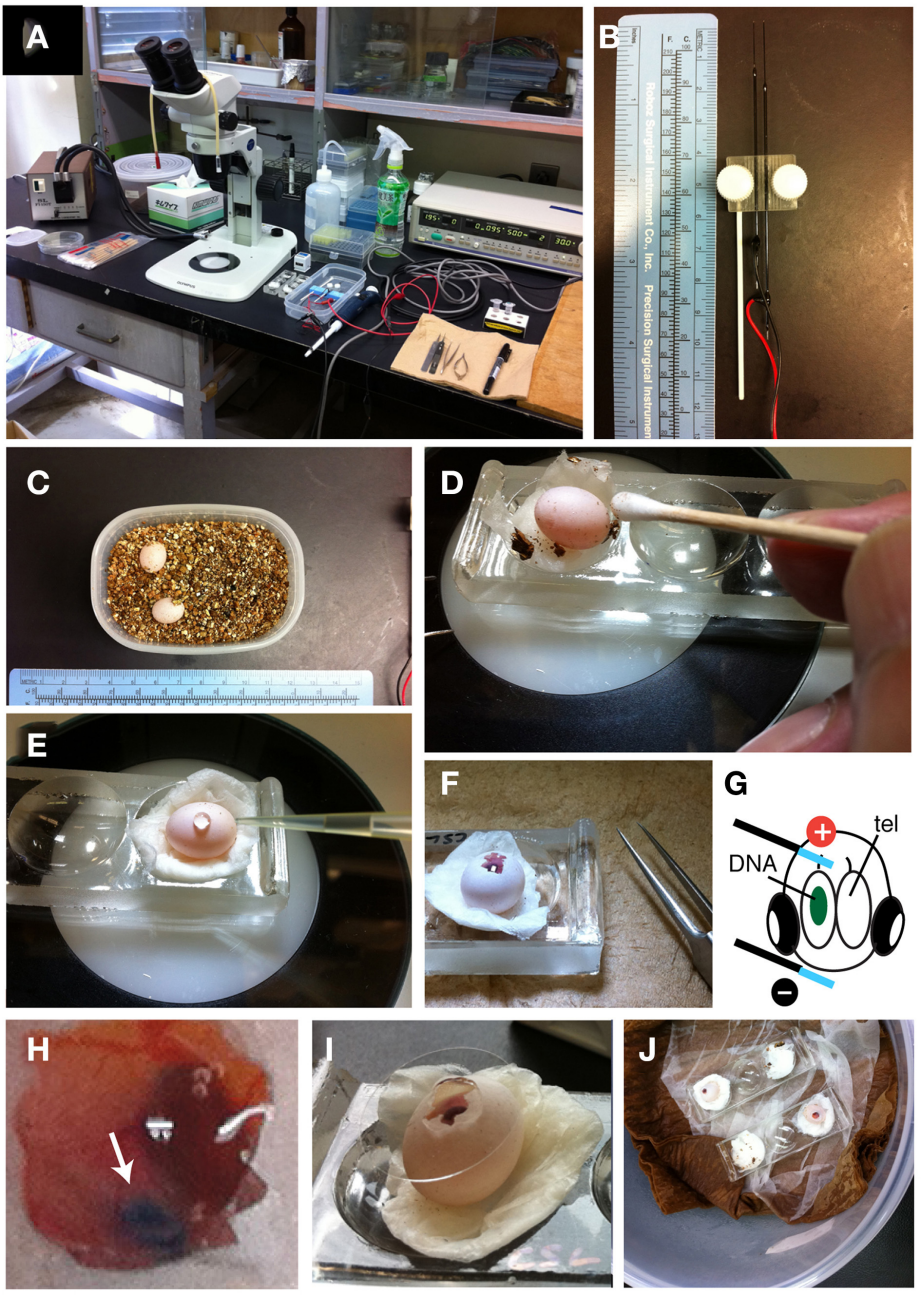
***In ovo* electroporation of gecko embryos. (A)** Experimental equipment. **(B)** Needle-type electrodes (CUY200S). **(C)** Two *P. pictus* eggs incubated in a small tapper with vermiculite. **(D)** Sterilization of the egg with 70% ethanol and a cotton stick. **(E)** HBSS was dropped through the hole of the shell. **(F)** The window was opened with fine forceps. **(G)** An illustration showing the position of the electrodes on the embryo. **(H)** High magnification of an electroporated embryo. Green-colored DNA solution was injected in the left lateral ventricle. **(I)** The window was sealed with a cover glass. **(J)** Incubation of operated embryos in the container.

Embryonic manipulation and electroporation are based on the procedures for gene transduction into developing avian embryos with slight modification (Figure 2) (Nomura et al., 2008; Nakamura, 2009). However, because the reptilian eggs are much smaller than chicken eggs, *in ovo* manipulation of reptilian embryos requires specific experimental techniques and training of surgical skills under the dissecting microscope (Figure 2A).

#### *In ovo* electroporation of gecko embryos

After oviposition, the laid eggs should be isolated from the mother animal to avoid accidental crushing of the eggs. In our laboratory, the eggs are immediately transferred to a plastic container filled with dried vermiculite (Figure 2C). To maintain embryonic respiration, small holes are made through the lid. The egg are approximately 10 mm in diameter and 12–13 mm in length (Figure 2C) (Noro et al., 2009). Fertilized eggs are incubated at 28°C in 50–60% humidity until manipulation. *In ovo* electroporation can be performed during 10–15 d.p.o. (days of post-oviposition); after these stages, the eyes and jaws increase rapidly in size, which make it difficult to access and electroporate to brains.

To begin *in ovo* electroporation, the egg is placed on a depression slide (Matsunami, Osaka, Japan) with moistened papers (Figure 2D, Prowipe, Elleair, Japan). The egg is sterilized with 70% ethanol and the surface of the shell was wiped with a cotton swab (Figure 2D, AspureAP-7, ASONE, Japan). Pieces of vermiculites attached to the eggs are removed at this step. The position of the embryo within the egg is confirmed by illuminating the egg with a fiber optic light (SL FI-150T, Sugihara Lab Inc., Japan). To open the shell, scratch the surface of the shell with fine forceps (VIGOR TW-705#5, B. Jadbow Inc, Switzerland) under a dissecting microscope (SZ61, OLYMPUS, Japan). Because the shell of gecko eggs is extremely fragile, care should be taken to open the shell with a forceps to avoid crushing the egg. After making a small hole in the shell, 50–100 μL of saline (HBSS: Hanks' buffered saline with the addition of 1% penicillin and streptomycin and 0.1% gentamycin) is added through the hole, and the further open by carefully removing the shell (Figure 2E). The vitelline and amniotic membranes were cut with microsurgical scissors (Figure 2F, RS-5620, ROBOZ, Germany). Next, 50–100 μL of HBSS is further added to the egg to maintain the space for embryonic manipulation.

To prepare the DNA solution for electroporation, purified plasmid DNA vectors are dissolved in sterilized phosphate buffered saline (PBS) with a non-toxic dye (0.01% fast green). Typically, we prepare 2.5–5 μg/μL of plasmid solution for the electroporation. Holding the head of the embryo with a fine forceps, the DNA solution is injected into the left or right side of the lateral ventricle with a fine glass capillary (MODEL G-1, NARISHIGE, Japan) that is connected to a mouth-pipette (Suction tube, Drummond, USA) or mini-injector (BJ100, BEX, Japan). Subsequently, needle-type electrodes (CUY200S, NEPAGENE, Japan) is inserted into the extra-embryonic space. Because DNA is negatively charged, a positive electrode was positioned at the target region (c.f., dorsal cortex), and a negative electrode was placed at the opposite side of the head (lower jaw; Figure 2G). The distance between the electrodes and embryos needs to be maintained (approximately 0.5–1 mm) to minimize the risk of tissue damage and hemorrhage by the direct application of electricity. Square waves of electric pulses (32 V, 50 ms, 950 ms interval, 2 or 4 pulses) are passed with an electric stimulator (SEN-3401, Nihon Kohden, Japan) or pulse generator (CUY21EDIT II, BEX, Japan). We compared survival rates of embryos at 48 h after electroporation and found that applying 4 pulses remarkably decreased the viability of gecko embryos (Table 1). To prevent microbe contamination, 50–100 μL of HBSS with antibiotics was applied into the extra-embryonic space. After the electroporation, the shell window was sealed with a micro cover glass (Figure 2I, 18 mm, #1, MATSUNAMI, Japan) attached with the tissue glue (1xHistoacryl L, B. Braun, Germany). The operated eggs were kept in a sterilized moist chamber (a plastic container with respiratory holes within the lid) and incubated at 30°C for 24 h to 1 month (Figure 2J).

**Table 1:** **Efficiency of the *in ovo* electroporation of the gecko embryos**.

**Stage (d.p.o.)**	**Number of embryos**	**Number of pulses**	**Number of survived embryos (2 days)**	^*^Electroporation efficiency (%)
12	2	2	0	N.D.
13	10	2	9	100
13	2	4	0	N.D.
14	18 (2)^**^	2 (2)^**^	11 (2)^**^	100
14	2	4	0	N.D.
16	4	2	2	100

* *Electroporation efficiency was determined by dividing the number of GFP-positive embryos by the number of collected embryos*.

** *Pilot experiments for the comparison of survival rates*.

#### *In ovo* electroporation of turtle embryos

**Figure 3:**
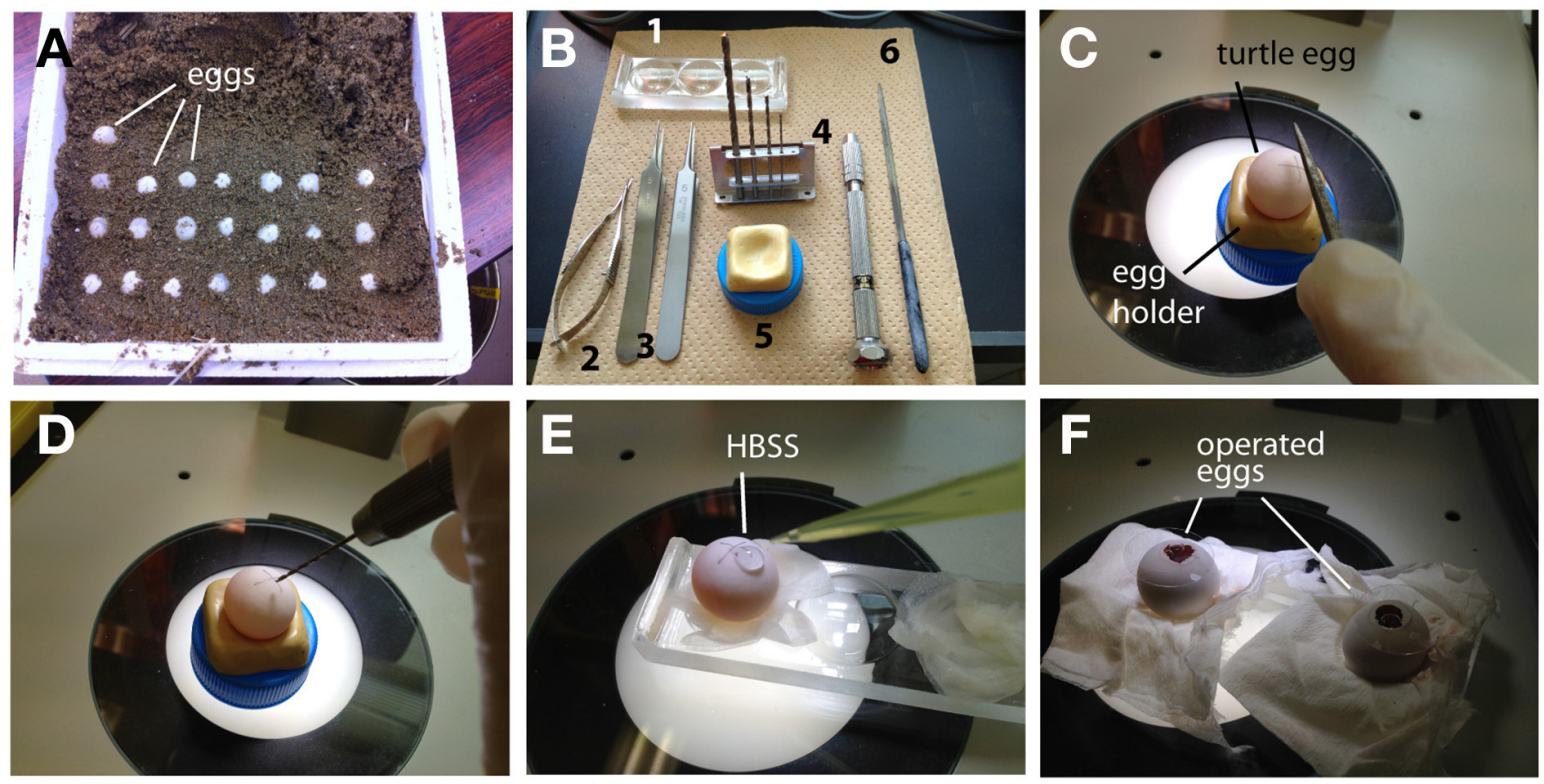
***In ovo*electroporation of turtle embryos. (A)** Turtle eggs in the delivery packet. Before transferring the eggs, the top of the shell was marked to maintain an upside-down orientation. **(B)** Tools for surgical manipulation. 1: A depression slide, 2: micro scissors, 3: forceps, 4: mini drill (pinvise), 5: hand-made egg stand, and 6: a metal file. **(C)** A small scar was made on the shell with a metal file. **(D)** A pin vise was used to drill the surface of the egg. **(E)** HBSS was dropped through the small window. **(F)** The window was sealed with a cover glass after electroporation.

*In ovo* manipulation of turtle embryos is similar to the method for gecko embryos with slight modifications. Because the early stages of the turtle embryos are tightly attached to the inside of the shell, frequent rotation of the egg will disrupt normal development of the turtle embryos. Thus, care should be taken to maintain the orientation of the egg after oviposition (Figure 3A). Fertilized turtle eggs are incubated in a highly moistened container at 28°C. We usually performed *in ovo* electroporation at stage 10–15 (10–15 days after fertilization).

The position of an embryo within the egg can be monitored by illuminating the egg with a fiber light. To open the turtle eggs, a small hole is made in the shell by drilling the top of the shell with a micro drill (0.5–0.8 mm in diameter, using a pin vise, TAMIYA, Japan) under the dissecting microscope (Figures 3B–D). After opening a small hole on the shell, 50–100 μl of HBSS with antibiotics was added through the hole, and the window was further widened by carefully removing the shell (Figure 3E). The chorion and amniotic membranes were cut with microsurgical scissors. After injecting a DNA solution (2.5–5 μl of DNA and 0.1% fast green in PBS) into the lateral ventricle, electroporation is performed with a needle-type electrode (CUY200S), and square pulses (32 V, 50 ms, 950 ms interval, 2 pulses) are applied to the target region of the embryos using an electric stimulator or pulse generator. After electroporation, the shell window was sealed with tissue glue and a micro cover glass as in the case of the gecko eggs (Figure 3F). The operated embryos are maintained in a moistened chamber and incubated at 30°C (Table 2).

**Table 2:** **Efficiency of the *in ovo* electroporation of turtle embryos**.

**Stage (TK)**	**Number of embryos**	**Number of pulses**	**Number of survived embryos (1 day)**	**Number of survived (2 day)**	^*^Electroporation efficiency (%)
13	19	2	14	9	100
15	6	3	3	3	33.3
16	5	2	3	3	0

* *Electroporation efficiency was determined by dividing the number of GFP-positive embryos by the number of collected embryos*.

#### *Ex ovo* culture of reptilian embryos

**Figure 4:**
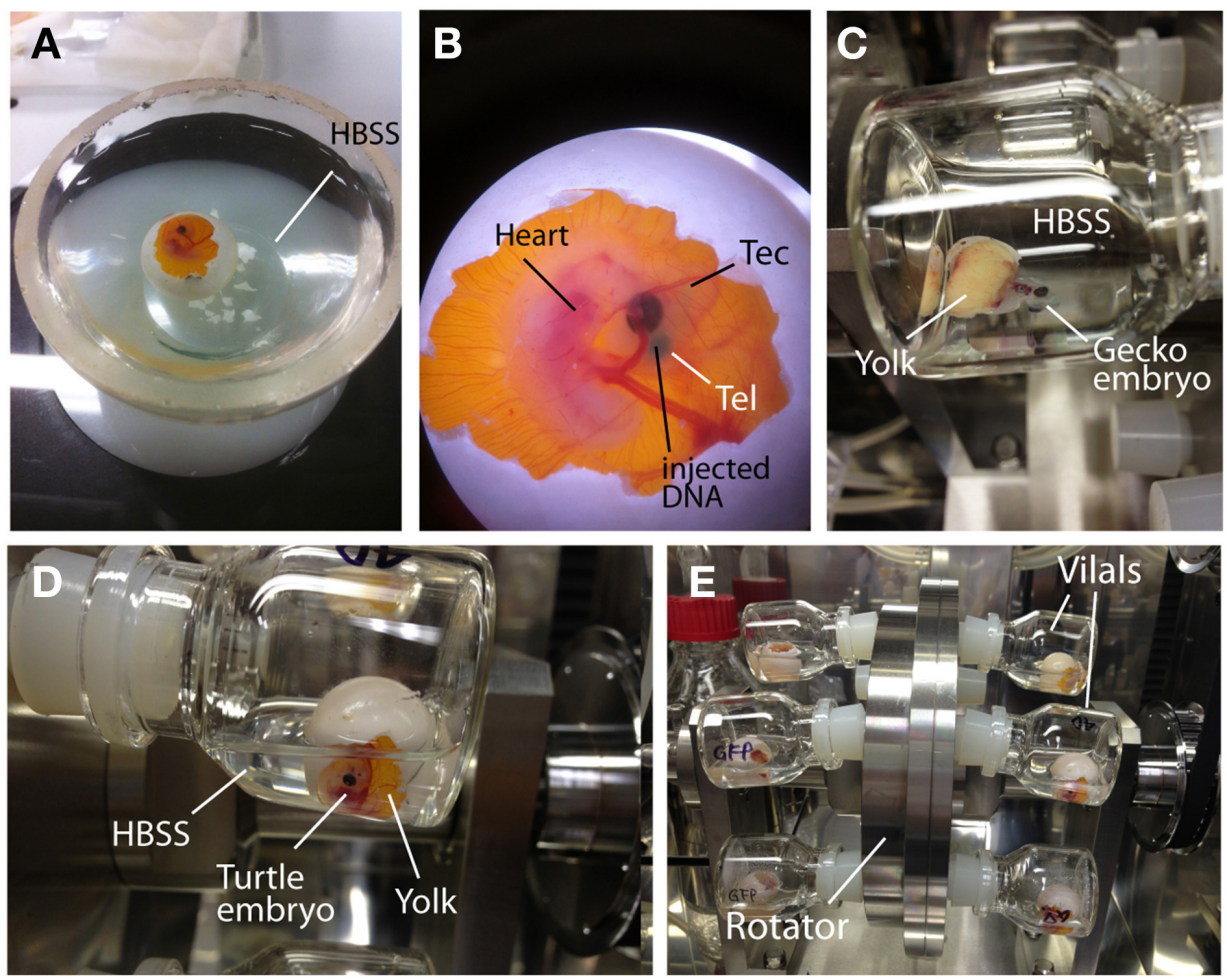
***Ex ovo* culture system for reptilian embryos. (A)** Turtle embryos were opened in HBSS. **(B)** A turtle embryo in which a DNA solution (green color) was injected into the lateral ventricle. **(C–E)** Incubation of gecko **(C)** and turtle **(D,E)** embryos in the whole embryo culture system. Electroporated embryos were cultured in glass vials filled with HBSS. Embryo containing vials were inserted into the rotator to supply oxygen continuously. To avoid crushing the embryos, the rotating wheel was not used during the culture.

Exposing the embryos from the shell to the medium dramatically facilitates accessibility to the embryos and increases the efficiency of electroporation (Buchtova et al., 2008; Tschopp et al., 2014). To allow embryonic development in the medium after electroporation, we established an *ex ovo* culture system for the middle stages of reptilian embryos (Figure 4 and Table 3). To begin *ex ovo* culture, fertilized gecko and turtle eggs are transferred to a glass evaporating dish filled with HBSS, and the shell was cracked within the medium with forceps to carefully expose the embryo from the extra-embryonic membrane (Figure 4A). The part of shell on the side of the yolk was kept to preserve the yolk sac (Figure 4B). Injection and electroporation can be performed within the evaporating glass (Figure 4B). After electroporation, the embryo was carefully transferred to a sterilized glass-made bottle (Ikemoto Rika, Tokyo, Japan) filled with 2 mL of HBSS with antibiotics (1% penicillin and streptomycin, 0.1% gentamycin) and cultured using the whole embryo culture system (Ikemoto Rika, Tokyo, Japan) in which oxygen is constantly supplied (95% oxygen, 5% carbon dioxide, 50 mL/min) to the embryos (Figures 4C–E). The culture temperature was maintained at 30°C to match ideal temperature for reptilian embryogenesis. Because the embryos are damaged by bottle rotation, the culture bottles were maintained in a static position during culture. The culture medium (HBSS) was replaced 24 h after starting the culture. The embryos can be maintained for approximately 2 days in this culture system because embryonic circulation was gradually decreases after 3 days of culture.

**Table 3:** **Efficiency of the *ex ovo* culture of gecko and turtle embryos**.

**Stage**	**Number of embryos**	**Number of survived embryos (24 h)**	**Number of survived embryos (48 h)**	^*^Electroporation efficiency (%)
Gecko d.p.o.8/9	3	3	2	100
Gecko d.p.o.15	1	1	1	100
Turtle TK16	6	6	2	100

* *Electroporation efficiency was determined by dividing the number of GFP-positive embryos by the number of collected embryos*.

### Expression vectors

Expression vectors designed for mammalian cells can be used for genetic manipulation in reptilian embryos. In general, the CAG promoter (cytomegalovirus enhancer with chicken ß-actin promoter) provides higher expression of transgenes in amniotic brains, particularly in the neural stem/progenitor cells (Niwa et al., 1991). We used several expression vectors, including pCAX-AFP (a variant form of GFP, Takahashi and Osumi, 2002) and pCAGGS-RFP (Nomura et al., 2008), which express fluorescent reporter proteins under the control of the CAG promoter. Expression vectors with Cre/loxP technology are useful for the restricted expression of transgenes in spatiotemporally controlled manners. The electroporation of Cre-recombinase expression vectors at a lower concentration (1 ng/μL) decreases the recombination frequency, which allows clonal labeling of neural stem/progenitor cells (Kato et al., 2010; Gotoh et al., 2012; Nomura et al., 2013a).

### Immunohistochemstry

To perform immunohistochemical analysis, embryos are fixed with standard fixative (4% paraformaldehyde in PBS) for overnight at 4°C and immersed in 20% sucrose for cryoprotection. The samples were embedded in OCT compound (Tissue-tek, SAKURA, Japan), and 14 μm of cryosections are made with a cryostat (LEICA CM1850, Germany). Several commercial antibodies are potentially applicable for immunohistochemistry in gecko and turtle embryos (Table 4) (Moreno et al., 2010, 2012), although not all the antibodies provide a single band with naïve brain extracts (**Figure 6** and our unpublished data).

**Table 4:** **The list of antibodies for immunohistochemistry of reptilian brains**.

**Antigen**	**Provider**	**Catalog no**.	**Dilution**	**Technical note**
Sox2	Abcam	ab97959	1:500	
Ctip2	Abcam	ab18465	1:500	
Satb2	Abcam	ab51502	1:500	
Foxp2	Abcam	ab16046	1:500	
Tbr2	Abcam	ab23345	1:500	TSA amplification
Tbr1	Millipore	AB2261	1:500	
ßIII-tubulin	Millipore	MAB1637	1:200	
Phospho- histon H3	Millipore	06-570	1:500	
Phospho- histon H3	Millipore	05-806	1:500	
NeuN	Millipore	MAB377	1:500	
DCX	Santa Cruz Biotech.	sc-8066	1:500	
Pax6	MBL	PD022	1:500	
Pax6^*^	DSHB	PAX6	1:500	Antigen retrieval with 2N HCl, 37°C, 15 min and TSA amplification
Rbpj-k	Cosmo Bio	SIM-2ZRBP2	1:500	Antigen retrieval with 2N HCl, 37°C, 15 min and TSA amplification

* *Specificity was examined with western blot in previous reports (Moreno et al., 2010, 2012)*.

### Representative results

**Figure 5:**
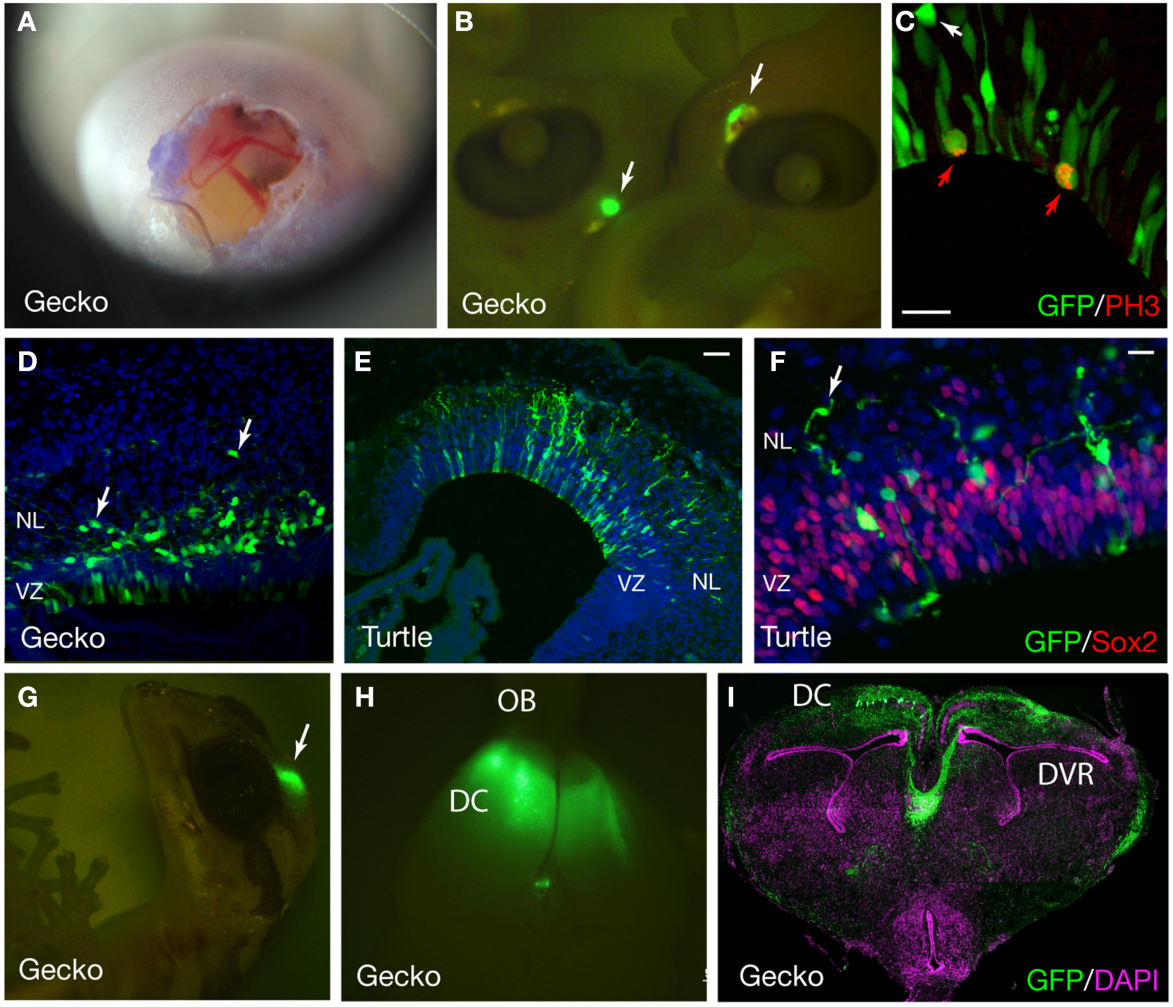
**GFP expression in the developing gecko and turtle cortex. (A)** Developing gecko embryo after electroporation. The image was captured using an iPhone4S camera through a magnifier. **(B)** Gecko embryos at 4 days after electroporation. GFP was expressed at the dorsal part of the telencephalon (arrows). **(C)** GFP expression in the cortical neural stem/progenitor cells. Mitotic GFP-positive cells were labeled with an anti-phospho histoneH3 (PH3) antibody (red arrows). A GFP-positive cell at the outside of the ventricular zone was not mitotic (white arrow). **(D)** The distribution of GFP-positive cells in the gecko cortex at 7 days after electroporation. Arrows indicate migrating neurons **(E,F)** GFP expression in the developing turtle cortex at 4 days after electroporation. Arrows indicate migrating neurons. **(G–I)** GFP expression in the gecko cortex at 1 month after electroporation. VZ, ventricular zone; NL, neuronal layer; OB, olfactory bulb; DC, dorsal cortex; DVR, dorsal ventricular ridge. Scale bars: 25 μm **(C,F)**, 50 μm **(E)**.

The expression of exogenous genes can be monitored soon after electroporation. We collected gecko and turtle embryos at several time points after the electroporation, and examined the expression of fluorescent reporters under a fluorescent microscope. At 2–4 days after electroporation, intense GFP expression was detected in the dorsal part of the gecko and turtle telencephalon (Figures 5A–C). Even at 1 month after electroporation, reporter fluorescence was still maintained in the brain (Figures 5G–I).

**Figure 6:**
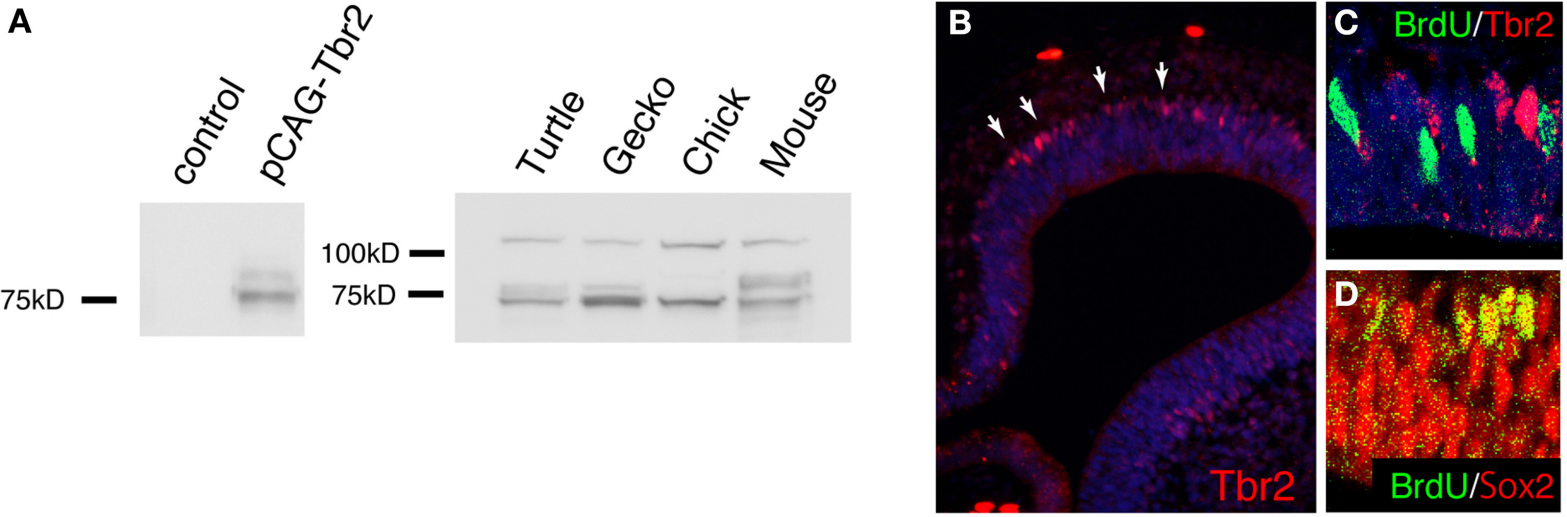
**The expression of Tbr2 in the developing gecko cortex. (A)** Western blot with anti-mouse Tbr2 antibody. The left panel shows lysate from HEK293 cells transfected with the expression vector for mouse Tbr2. The control lane was whole cell lysate without transfection. A major band was detected at the predicted molecular weight (72 kD) for mouse Tbr2. A slightly lager band was possibly due to post-translational modification. The right panel shows western blot of embryonic turtle (st17), gecko (d.p.o.18), chick (E7), and mouse (E14) brain lysate. Together with the bands of predicted molecular weight (72 kD), additional larger bands were detected in all examined species. **(B–D)** Immunohistochemistry of the developing gecko cortex (d.p.o. 18) with anti-Tbr2 antibody. Tbr2-positive cells were detected at the basal side of the ventricular zone (white arrows). **(C)** Tbr2-positive cells did not overlap with BrdU-incorporated cells. **(D)** All BrdU-incorporated cells were Sox2-positive. Detailed immunohistochemistry and BrdU incorporation protocols were described previously (Nomura et al., 2013a).

At 2 days after electroporation, GFP expression was exclusively detected in mitotic neural stem/progenitor cells that were localized at the ventricular zone of the developing gecko cortex. These neural stem/progenitor cells have a radial fiber similar to the mammalian radial glial cells, but the fibers extend in a curved manner at the neuronal layer as in the case of avian cortical radial fibers (Nomura et al., 2008, 2014). At 4 days after electroporation, GFP-positive cells migrated from the ventricular zone and positioned at the marginal zone (Figures 5C,E,F). GFP-positive migrating neurons in the developing gecko cortex exhibited multipolar morphology, similar to intermediate progenitor cells (IPCs) in the subventricular zone (SVZ) of the mammalian neocortex (Miyata et al., 2004; Noctor et al., 2004; Englund et al., 2005). However, unlike mammalian IPCs, we could not detect mitotic activity in the GFP-labeled multipolar cells in the gecko cortex (Figure 5C). This result is consistent with our observation that Tbr2-positive cells in the developing gecko cortex are post-mitotic neurons (Figure 6) (Nomura et al., 2013a). At 1 month after electroporation, GFP-expressing cells were still detected in the medial and dorsal cortex of gecko embryos (Figure 5G). Notably, these GFP-positive cells exhibited reptilian-type pyramidal neurons and extended axonal fibers toward the contra-lateral side of the cortex, which constitutes the pallial commissure in reptiles (Figures 5H,I).

**Figure 7:**
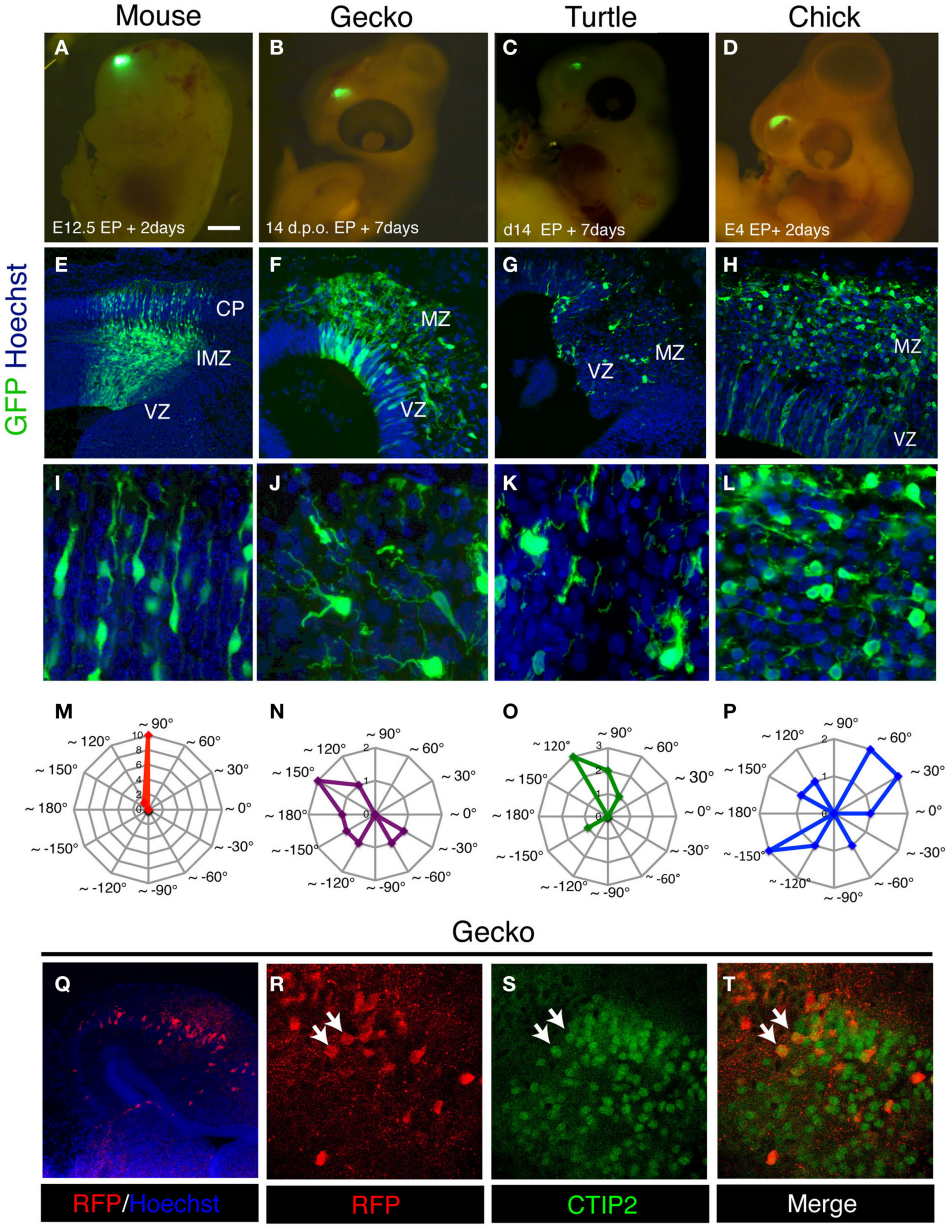
**Characteristics of migrating neurons in the developing amniote pallia. (A–D)** Electroporation of GFP-expression vector into the developing mouse **(A)**, gecko **(B)**, turtle **(C)**, and chick **(D)** pallia. **(E–L)** Distribution and morphology of GFP-positive migrating neurons in the mouse neocortex **(E,I)** and the gecko **(F,J)**, turtle **(G,K)** and chick **(H,L)** pallia. **(M–P)** Contour graphs of the longest process orientation of mouse (**M**; the data were taken from the cortical plate), gecko **(N)**, turtle **(O)**, and chick **(P)**. The angles of the processes were calculated against the ventricular plane. Each contour line represents the number of cells. **(Q–T)** The expression of CTIP2 in RFP-positive pallial neurons in the developing gecko cortex (white arrows in **R–T**).

Recent studies have shown that the transition from the multipolar to bipolar shape in the migrating neurons is critical for the development of mammalian neocortex (Noctor et al., 2004; Hand et al., 2005; Heng et al., 2008; Ohtaka-Maruyama et al., 2013; Kawauchi, 2014; La Fata et al., 2014). In contrast to the mammalian neocortex, migrating neurons in the developing gecko and turtle cortex still maintained multipolar morphology at 7 days after electroporation (Figures 5D, 7A–L). To quantify the orientation of leading process in migrating neurons, the angle of the longest process in each neuron relative to the ventricular surface was quantified in mouse, gecko, turtle and chicken cortex/dorsal pallium. Comparison of leading process orientation demonstrated that all migrating neurons in the mammalian cortical plate are vertically aligned: thus, all neuronal processes are directed to the pial surface. In contrast, migrating neurons in the reptilian and avian marginal zone are not tightly aligned and extend leading process in various directions (Figures 7M–P). Thus, the strict alignment of bipolar migrating neurons in the cortical plate is a unique characteristic in the developing mammalian cortex. However, we also confirmed that the expression of mammalian cortical plate markers, such as Tbr1, CTIP2, and SATB2, is also detected in the developing gecko cortex (Figures 7Q–T) (Nomura et al., 2013a), suggesting that some of the molecular characteristics of the cortical plate neurons are conserved between the mammalian and reptilian cortex.

## Discussion

Comparative analyses of extant amniote brains are powerful approaches to understand the evolutionary processes of the mammalian neocortex and homologous structures in non-mammalian lineages (Molnar et al., 2006; Aboitiz, 2011; Medina et al., 2013). Previous histological studies revealed that the stellate morphology of migrating neurons in the developing reptilian cortex resemble migrating neurons in the early stages of mammalian neocortex (Goffinet, 1983). Based on the ontogenic analyses, Marin-Padilla hypothesized that mammalian neocortex has dual origins: the superficial and deepest neurons (layer I and IV) retain ancestral phenotypes that are reminiscent of the amphibian or reptilian cortex, whereas the later-born cortical plate neurons (layer II-V) are recently acquired during mammalian evolution (Marin-Padilla, 1971, 1978). Our *in vivo* cell tracing analyses indicated that (1) multipolar neurons in the reptilian cortex do not exhibit mitotic activity and (2) multipolar-to-bipolar transition of migratory modes is not detected during the reptilian corticogenesis. These data support the idea that both amplification of IPCs (Martinez-Cerdeno et al., 2006; Cheung et al., 2007; Charvet et al., 2009; Puzzolo and Mallamaci, 2010) and unipolar cortical plate neurons with a “locomotive mode” are derived developmental processes in the mammalian neocortex (Aboitiz et al., 2001), through which the expansion of neuron numbers and multiple laminar structures evolved. However, the morphological similarities of migrating neurons are not always associated with common cellular dynamics and gene expression patterns. Thus, the reptilian neurons are not simply equivalent to the early stages of mammalian cortical neurons or ancestral neuronal subtypes.

In addition to cell tracing of migrating neurons, we applied several developmental techniques to analyze reptilian corticogenesis, such as (1) lineage tracing of neural stem/progenitor cells, (2) quantification of reporter activities for signaling molecules, and (3) gain- and loss-of-function analyses of specific genes in the developing reptilian cortex (Nomura et al., 2013a). These experimental approaches unveiled further unique characteristics of reptilian neural stem/progenitor cells. For example, the rates of proliferation and differentiation of reptilian cortical progenitors are very slow and contribute to the production of a lower number of cortical neurons. Some of these characteristics depend on Notch signaling, and experimental manipulation of a Notch downstream effector dramatically increased neuronal production in geckoes. We hypothesized that after the diversification of mammalian and non-mammalian amniote lineages, some critical changes in neural stem cell regulation might have occurred in the ancestral mammals and thus provided the expansion of cortical areas and massive generation of excitatory neurons (Nomura et al., 2013a,b, 2014).

Recently, whole genome sequences of Chinese softshell turtle and sew turtle have been performed, which have confirmed that turtles must be positioned phylogenetically in archosaur groups in amniotes (Wang et al., 2013). The data also demonstrated that conserved and derived genetic programs in turtle embryogenesis contributed to the evolution of the turtle-specific body plan (Wang et al., 2013). Although genome analyses of Madagascar ground gecko have not been accomplished, draft genomes of green anole lizard (*Anolis carolinensis*), a related species to the gecko, have been published (Alfoldi et al., 2011). Genomic information of the Anolis lizard revealed unique characteristics in its genomic composition, such as homogenization of the GC content and higher number of mobile elements than other amniotes (Alfoldi et al., 2011). Additional studies of the comparative genomics of reptiles will clarify how genetic and epigenetic changes contributed to brain evolution in distinct lineages of amniotes. Genomic sequences of the Chinese softshell turtle and Anolis lizard are available at the website of the Ensemble Genome Browser (http://asia.ensembl.org/index.html).

Currently, we have only successfully performed *in ovo* electroporation during a narrow window of time (d.p.o. 10–16 for gecko embryos and stages 10–15 for turtle embryos). Because reptilian eyes and jaws rapidly increase in size during embryogenesis, positioning the electrodes to target dorsal cortex is technically difficult at later embryonic stages. Application of an *ex ovo* culture system for gecko and turtle embryos is also limited for 3–4 days, most likely due to the lack of some essential nutrients and/or sufficient oxygen supply. Further improvements of gene delivery tools and/or culture conditions are required to manipulate embryos at any developmental stage.

Electroporation with a transposon-mediated genomic integration system provides permanent lineage tracing in mammalian and non-mammalian vertebrates (Garcia-Moreno et al., 2014; Loulier et al., 2014). Furthermore, recent advances in genome editing tools, such as TALEN (transcription activator-like effector nuclease) and CRISPR/Cas (clustered regulatory interspaced short palindromic repeats/CRISPR-associated proteins), extend the possibility of genetic manipulation in a variety of organisms (Aida et al., 2014; Kaneko et al., 2014; Pal et al., 2014). The *in vivo* delivery of CRISPR/Cas vectors induces direct somatic recombination in target tissues, which enables the site-specific mutation of endogenous genes (Xue et al., 2014; Yin et al., 2014). The application of these new research strategies to the study of comparative brain development provides a new avenue for the understanding of the origin and evolution of amniote brains, particularly the mammalian cerebral cortex.

### Conflict of interest statement

The authors declare that the research was conducted in the absence of any commercial or financial relationships that could be construed as a potential conflict of interest.
